# Does Leader–Member Exchange (LMX) Ambivalence Influence Employees’ Constructive Deviance?

**DOI:** 10.3390/bs14010070

**Published:** 2024-01-19

**Authors:** Zhen Liu, Qunying Liu

**Affiliations:** School of Management, Shanghai University, Shanghai 200444, China; zhenliu@shu.edu.cn

**Keywords:** ego depletion, employees’ constructive deviance, leader–member exchange ambivalence, role-breadth self-efficacy

## Abstract

The ambivalent experience of superior–subordinate relationships is widespread in organisations and has gradually become an important factor influencing employees to actively engage in extra-role behaviours. However, employees’ constructive deviance is extremely important for organisational development as they are important extra-role behaviours for organisational innovation and change. Owing that academic research on the antecedents of employees’ constructive extra-role behaviours has lacked attention to individual emotional variables such as the leader–member exchange ambivalence, by drawing on self-control resource theory and social cognitive theory, this study examined the effects of leader–member exchange ambivalence on employees’ constructive deviance, as well as the role of ego depletion and role-breadth self-efficacy. Based on a two-point questionnaire survey of 332 employees from different industries in China, the study tested hypotheses with SPSS 27 and AMOS 27 and found that the more leader–member exchange ambivalence, the less likely they were to engage in employees’ constructive deviance, leader–member exchange ambivalence affected employees’ constructive deviance through ego depletion, and when role-breadth self-efficacy is high, the lower the ego depletion of employees with leader–member exchange ambivalence, the more likely they are to engage in employees’ constructive deviance. This study is intended to guide organisations to pay attention to the problem of individual internal conflict arising from superior–subordinate relationships, to remove the barriers to constructive transgression by individuals, and to truly exploit the innovative capacity of individual organisations. The study suggests that managers should pay attention to the negative effects of employees’ perceived ambivalent experiences of supervisor-subordinate relationships, maintain consistency, and build positive social exchange relationships with their employees. Organisations should strengthen the training of leaders and employees to eliminate the serious internal attrition that organisations face from social network relationships. And employees should face the limitations of resources and reduce dependence on the leader–member exchange relationship as the dependence for their work attitudes and behaviours.

## 1. Introduction

In the current VUCA era, innovation is occurring at an unprecedented pace, enhancing global productivity and production efficiency, and ultimately improving the quality of life for people worldwide. Innovation capability is a crucial indicator of a country’s comprehensive national strength, enabling it to seize opportunities and gain an advantage in the intense international competition. According to the China Association of Listed Companies’ data, over a hundred typical cases of successful digital transformation by listed companies were recorded in 2023 [1]. In contrast, the global economy demonstrated a trend of contraction in 2022, resulting in the closure of more than 3000 companies from China, including unicorn companies and industry giants. During a period of widespread closures, public attention is frequently drawn to the declining market demand, limited innovation and a one-dimensional business model of the company. This presents a range of challenges, as the company is constrained by its internal development model and operational structure, making it harder to adjust course [2]. Even when the business is grappling with external risk pressures, internal management risks remain an ongoing concern. Hence, adhering to conventional thinking to surpass limits and challenging established practices has now become the primary goal for modern enterprises that strive for innovation and development. This cannot be achieved without employees’ recognition of breaking norms, taking risks, and being innovative in their proactive actions.

Employees’ resistance to proactive behaviours may be due to the depletion of psychological resources developed in a negative work environment. According to the 2022 Gallup World Poll, 44% of workers from over 160 countries reported experiencing significant stress the previous day, with over 20% reporting significant feelings of anger or sadness [3]. The 2023 Gallup Global Workplace Environment Report reveals that 59% of employees are leaving their jobs without making their reasons known. Of those who do, 41% cite issues with engagement or culture, such as a lack of recognition for contributions, limited opportunities for advancement, and unapproachable managers [4]. Organisations should recognise that workplace conditions, including human relationships, significantly impact individual happiness. According to psychologist Mindy Shoss, organisations must invest time and effort to rebuild trust and reduce uncertainty and fear of the future, as people’s jobs and lives can change suddenly. Multiple studies conducted by the American Psychological Association have shown that employees are more likely to express their opinions, generate more creative and innovative solutions, and engage more in quality improvement efforts when they believe they can take more risks without facing negative consequences. This highlights the importance of creating a culture that encourages risk-taking and values diverse perspectives.

Employee acceptance and adherence to organisational norms are widely recognised as fundamental requirements for enterprises to maintain healthy operations and achieve their goals [5]. However, the swift transformations in digital technology and industrial revival have rendered certain internal norms insufficient in fostering sustainable progress within enterprises [6]. Additionally, the excess number of organizational layers, low management level, and outdated organizational framework are major factors of lag [7]. In recent years, the sudden onset of the epidemic has had a significant adverse effect on China’s real economy, and the shortcomings of enterprise management have gradually become apparent. A “rule-oriented” management approach impairs organisational efficacy and hampers employee adaptability and innovation [8]. Amidst a complex and turbulent environment, leaders are endeavouring to encourage employees to engage in constructive transgressions for the purpose of advancing organisational change and innovation [9]. However, there is a prevalent phenomenon of “can-don’t-do” impeding organisations from salvaging themselves, where competent employees are hesitant to undertake actions outside their work that benefit the organisation. This includes “Employees’ Constructive Deviance” that breach organisational norms in favour of the organisation or its members [10].

Recent academic research on the antecedents of employees’ constructive deviance primarily focuses on the individual and situational factors [11]. In recent years, researchers have increasingly investigated various leadership styles and contextual factors, including self-sacrificing leadership and authentic leadership [9,12]. The majority of studies concentrate on the factors that stimulate positive nonconformist behaviours, disregarding the barriers that hinder such behaviours and paying insufficient attention to specific emotional factors [13]. Relationships play a pivotal role in steering employee behaviour within organisational social networks, with changes in the quality of the associations between employees and their leaders [14] inducing alterations in personal emotional states which should not be overlooked. There has been extensive online discourse surrounding leaders who exhibit varying degrees of competence and incompetence, distribute resources and time disparately, and display erratic behavior. The core issue is that employees encounter “the contradictory experience of superior-subordinate relationships [14]” and “the lack of self-control generates negative emotions [15]” as two primary causes for hindering work autonomy and individual effectiveness. In turn, this causes issues such as the bottleneck of corporate change and inadequate motivation for innovation. Therefore, in the face of the complex and ever-changing external market, companies must maximize the creativity and flexibility of their employees to achieve sustainable growth in the fierce competition [8]. The creation of new development potential in an enterprise necessitates the unified contributions of all individuals within it to utilise their knowledge and strengths. As a result, innovative solutions and changes that defy traditional conventions can serve as a crucial means for numerous enterprises to enhance management efficacy and consequently attain lasting progress. It is insufficient to rely solely on breaking rules for innovation. Instead, we must encourage employees’ constructive deviance that is more extensive in scope than just innovating through rule-breaking. This will enable us to effect a complete shift from individual to departmental levels, and ultimately to the entire organisation, thereby achieving significant qualitative advancement.

Therefore, this study aims to explore how to enhance both individual and overall effectiveness in organisations where the phenomenon of unproductive individuals exists. From the perspective of superior–subordinate relationships, the study introduced the self-control resource theory to examine the mechanisms for inhibiting employees’ constructive deviance, an extra-role activity. Additionally, the study incorporated the social cognitive theory to investigate whether employees with varying levels of role-breadth self-efficacy traits can successfully avoid or mitigate emotional disturbances resulting from leader–member exchange ambivalence, potentially facilitating greater participation in employees’ constructive deviance.

Data were obtained through a questionnaire survey, and a mechanism model was constructed to examine how leader–member exchange ambivalence affects employees’ constructive deviance from both emotional and rational perspectives. The analysis included 332 samples of corporate employees from various industries, and research hypotheses were subsequently tested. Key innovations presented in this study include that the study first integrates affective and cognitive factors in a single model. It explores the influence of leader–member exchange ambivalence on employees’ constructive deviance. This enriches research on the outcomes of such experiences and expands research on the antecedents of employees’ constructive deviance among employees. Secondly, this study combines the resource theory of self-control and the theory of social cognition, enhancing the connection between the two theories. Additionally, the study carries significant practical implications. Examining the adverse effects of superior–subordinate relationships on employees through the lens of ego depletion permits companies to recognize that prevailing internal conflicts significantly impede the company’s growth and progress. Additionally, it helps managers identify the root causes and devise effective management strategies.

## 2. Literature Review

### 2.1. Hypotheses Development

#### 2.1.1. Leader–Member Exchange Ambivalence and Employees’ Constructive Deviance

Ambivalence is common within individuals in organizations. Various types of ambivalence have been studied, including trait ambivalence [16], expressive ambivalence [17], emotional ambivalence [18], attitudinal ambivalence [19] and relational ambivalence [20]. Relational ambivalence refers to the coexistence of positive and negative social network relationships. Inconsistent experiences, particularly those perceived from the leader–member exchange relationship, become increasingly important [21]. With the progression and intensification of the leader–member exchange relationship from absolute to relative and from static to dynamic, scholars have increasingly directed their attention towards examining ambivalence within superior–subordinate relationships, which is both relative and dynamic. Lee et al. initially introduced the concept of leader–member exchange ambivalence, which refers to a subjective experience in which an employee has both positive and negative thoughts towards superior–subordinate relationships [22]. For instance, employees may perceive their leaders as aligning with their interests at times, while also perceiving that leaders abstain from leveraging their authority to address difficulties for them.

Based on the Borich Needs Assessment Model, it has been found that the most important skill closely related to innovation is interpersonal relationship management [23]. Social skills promote both teamwork and improved interaction between parties. Both enhance organisational innovation by facilitating the exchange of ideas and the sharing of information. Therefore, the role of interpersonal relationships is important for constructive employee transgression. Individuals in organisations with good relationship management skills are able to take the interpersonal risks associated with breaking organisational rules and are willing to contribute to creative change. Individuals with less experience of conflicting hierarchical relationships are also willing to share their ideas and find creativity in their work [24].

Previous research has indicated that relationships between superiors and subordinates, as a significant context variable for measuring the level of “insiders,” directly affect employee behaviour within organisations and serve as a crucial tool for decoding extra-role behaviours [25]. When the quality of the relationship is weak, employees are more likely to perform only the tasks specified in their job responsibilities [26]. In contrast, in a high-quality relationship, employees are more inclined to take responsibility for the development of the organisation, address prevailing issues, and provide positive recommendations [27]. Such a positive work environment activates their own inherent ability to drive change, resulting in a stronger impetus towards constructive action [28]. Studies investigating the impact of ambivalence in hierarchical relationships have consistently reported that if employees experience ambivalence stemming from the leader–member exchange relationship, it can adversely affect their well-being, behaviour, and performance [14,29,30]. Furthermore, it was reaffirmed that the leader–member exchange ambivalence impedes the manifestation of proactive and nonethical pro-organisational conduct by employees [31]. The influence of such experience on employees’ constructive deviance of employees warrants extensive investigation. Leaders’ time and resource limitations have resulted in the widespread incidence of employee-perceived leader–member exchange ambivalence [14]. The requirement for leaders to balance close relationships and hierarchical differences with their subordinates [32] leads to employees perceiving both positive and negative experiences from the superior–subordinate relationship. Such perception may hinder the occurrence of voluntary behaviours. For instance, leader–member exchange ambivalence may reduce employees’ proactive behaviours by lowering their perceived organisational status [14]. When ambivalence elicits negative emotions, employees refrain from participating in organisational citizenship behaviours and may even engage in counterproductive behaviours [33]. Furthermore, ambivalent experiences undermine an individual’s cognitive coherence, dissuading them from pursuing goal-directed actions, and instead, leading them to blame or excessively ruminate, triggering anxious reactions and avoidance tendencies. This, in turn, results in paralysis and resistance to change [34]. However, employees’ constructive deviance involves deliberately taking risks to challenge organisational norms and leadership authority with the aim of effecting organisational change. When leaders inspire trust among subordinates, they, in turn, invest more effort towards maintaining the relationship. This includes engaging in organisational citizenship behaviours [35], constructive advice behaviours [36], which are critical aspects of employees’ constructive deviance. When leaders fully empower their subordinates, it enhances their sense of ownership and encourages them to take responsibility for building the organisation. As a result, they engage in employees’ constructive deviance [37]. The trust bestowed by leaders reinforces employees’ motivation towards employees’ constructive deviance and allows them to receive more support from external resources in the face of implementing risky behaviours. If employees perceive that their superior treats them fairly, even in difficult situations, they are more likely to engage in employees’ constructive deviance. However, if they lose trust in their leader, they may reduce these behaviors. Based on these, hypothesis H1 was established.

**H1.**  *There is a negative impact of leader–member exchange ambivalence on the employees’ constructive deviance*.

#### 2.1.2. The Mediating Role of Ego Depletion

Self-control resource theory explains the mechanism of self-control failure from a resource depletion perspective. It has been widely noticed and validated in the fields of social psychology and organisational behaviour. Previous studies have examined self-defeat from a resource depletion perspective. Focusing on direct research on the allocation and depletion process of self-control resources further enhances the explanatory power of self-defeat for employee behaviour in the workplace [38]. Social psychological research indicates that individuals require limited self-control resources to perform self-control tasks [39]. Additionally, individual goal-directed behaviours, such as maintaining and controlling safety, also rely on self-control processes [40]. Therefore, individuals who regulate their attention and emotions experience a significant reduction in self-control, known as the phenomenon of “ego depletion”.

The experience of ambivalence in superior–subordinate relationships can have a significant impact on employees’ emotions and attitudes. According to the theory of ambivalence amplification, individuals’ cognitive responses are amplified with ambivalence recognition, resulting in extremely negative or positive attitudes [41]. The inconsistent sequencing of leadership may motivate individuals to engage in ambivalent identification, resulting in negative perceptions of reciprocity [42]. Individuals with ambivalent experiences of leader–member exchange relationships tend to be highly sensitive to the relationships of others [43,44,45], consuming significant psychological resources and generating negative emotions due to a lack of trust. Individuals with high ambivalent cognitions are prone to cognitive dissonance, which can lead to negative interpretations of superior–subordinate relationships [46]. This, in turn, can decrease work well-being [29].

The theory of resource depletion in self-control suggests that when individuals regulate their behaviour [47], their limited resources are depleted, resulting in ego depletion. Ambivalent experiences can cause discomfort or distress and require psychological resources to cope with in hierarchical relationships [18]. As people have restricted resources available for self-control activities, for instance concentration and attention, partaking in self-control behaviours, such as eliminating ambivalent experiences, may lead to affective depletion or emotional exhaustion. When individuals experience fatigue, their attention, outputs and behavioural performance on subsequent tasks are somewhat reduced [48,49,50]. Nonetheless, leaders within the workplace often encounter limitations of both time and resources, which results in an inability to treat all employees equally. Occasionally, employees may question the standard of communication between leaders and themselves, leading them to become excessively entangled in psychological and ideological conflicts, thus hindering their ability to concentrate on their responsibilities. Based on the Self-control Resource Theory, depletion of psychological resources may lead to the “ego depletion” effect among employees, meaning a decline in self-control resources and reduced effectiveness in self-executive functions [51]. In this respect, hypothesis H2 was established.

**H2.**  *Leader–member exchange ambivalence significantly and positively correlate with employees’ ego depletion*.

Research has shown that ego depletion can have negative effects on cognitive, affective, and volitional behaviours. According to Fischer et al., ego depletion can lead to a depletion of psychological resources, resulting in changes in cognitive levels, such as pessimism and doubts about one’s abilities [52]. Additionally, ego depletion can influence an individual’s perception of risky decision-making to some extent. Price and Yates conducted a study in which participants were assigned to either a depletion task or a nondepletion task [53]. The results showed that the group assigned to the depletion task tended to choose less-challenging topics when selecting test questions. This suggests that after experiencing ego depletion, individuals tend to adopt a more cautious approach and make less-risky decisions. Additionally, research has shown that individuals experiencing ego depletion are more susceptible to anxiety and have reduced emotional control [54]. However, according to Zhang et al., employees’ levels of ego depletion can be reduced through positive thinking interventions, leading to increased work engagement [55]. Furthermore, research has demonstrated that ego depletion can hinder the development of relationships [56]. Additionally, an individual’s ego depletion can influence others’ perceptions of their trustworthiness and subsequently impact the establishment of a trusting relationship [57]. It is important to note that subjective evaluations have been excluded from this analysis. In recent years, the field of organisational behaviour has increasingly focused on ego depletion. Studies have shown that workplace behaviours, including burnout [58], pro-social behaviour [59,60], counterproductive behaviour [33], and ethical advice [61], are influenced by ego depletion.

According to the energy–motivation pathway of the proactive motivation model, positive emotions assist individuals in establishing challenging objectives and confronting ambiguity to carry out proactive behaviours [62]. Employees, when in a positive mood, undertake creative problem-solving and execute constructive deviance to attain mutually beneficial outcomes for both the person and the organisation [63]. Furthermore, psychologically resilient individuals are better equipped to withstand interpersonal stress and work-related risks associated with constructive transgressions, thereby increasing the likelihood of such transgressions [12]. However, employees in a negative mood evaluate and perceive their work time and environment negatively [33], forming critical evaluations of their organisation and colleagues. Subsequently, this reduces their motivation to voluntarily assist their organisation and colleagues and ultimately weakens their ability to engage in extra-role behaviours [64]. Based on the opinions stated above, hypothesis H3 was established.

**H3.**  *There is a negative correlation between employee ego depletion and employees’ constructive deviance*.

The ego depletion theory suggests that constructive behaviours can result in varying degrees of ego depletion for individuals [65]. When individuals identify existing problems or propose innovative ideas to improve organisational functioning, they may experience anxiety and ego depletion due to concerns about the negative consequences of transgressive behaviours [66]. In situations where self-control resources are severely lacking, individuals may deplete their psychological resources during hierarchical conflict. This may leave them without sufficient psychological resources to withstand the consequences of transgressive behaviours. It has been suggested that engaging in tasks, transgressive behaviours, and civic actions can result in ego depletion. When individuals lack resources for self-control, they become more impulsive and prone to transgressive tendencies [67]. They tend to select behaviours that provide immediate benefits but lead to higher costs in the long term [38]. When negative emotions deplete an individual’s ego resources, they may lack the resources needed for constructive deviance. In fact, they may even engage in destructive transgressions. Research has long established that the number of ambivalent relationships independently predicts psychological distress, and that negative emotions mediate one’s emotional responses in situations when ambivalence arises due to interpersonal relationships [68]. Negative emotions were found to have a significant mediating role in explaining the adverse effects of ambivalence in superior–subordinate relationships on task performance [22]. In general, leader–member exchange ambivalence increases uncertainty in the employee-leader relationship, lowers the employee’s faith in the leader, and leads to an adverse interpersonal evaluation of the leader. When employees experience ego depletion, they are less likely to engage in transgressive behaviours that benefit the organisation. Just like the confirmed leader–member exchange ambivalence can hinder transgressive innovations by increasing job anxiety [69], the leader–member exchange ambivalence can also impede spontaneous employees’ constructive deviance that benefits the organisation by causing ego depletion in employees. Therefore, the present study postulates the following hypothesis H4.

**H4.**  *Ego depletion acts as a partial mediator of the association between leader–member exchange ambivalence and employees’ constructive deviance*.

#### 2.1.3. The Moderating Effect of Role-Breadth Self-Efficacy

According to Social Cognitive Theory, self-efficacy refers to an individual’s assessment of their ability to complete a given activity [70]. A strong sense of efficacy can enhance positive psycho-cognitive motivation, leading to increased self-confidence and improved control when evaluating the outcome of their actions. Role-breadth self-efficacy is the perception of an employee’s ability to perform a wider range of proactive work tasks beyond the specific technical requirements of their job. This refers to employees who are not content with their current job and take the initiative to take on greater responsibility for out-of-work roles, with the confidence that they can tackle more roles [71]. Compared to self-efficacy, role-breadth self-efficacy is broader in scope and perceived meaning.

The interaction between individuals’ cognition, environment and behaviour can determine employees’ behavioural motivation [72]. Previous research has demonstrated that ego depletion negatively impacts employees’ constructive deviance, although certain situational variables may weaken this effect. Positive psychology, such as role-breadth self-efficacy, has been linked to employees’ proactive and innovative behaviours [73]. Employees with high self-efficacy in a broad role tend to undertake proactive extra-role behaviours to achieve their goals and derive value beyond their respective domain [74]. It is worth noting that self-control resources are not constant as individual differences exist, and they can be depleted. Under conditions of high role-breadth self-efficacy, employees’ personal positive psychological cues and strong role expectations can alleviate the extent of ego depletion, assisting them in exiting negative states and subsequently taking the initiative to make beneficial constructive deviance for the organisation. Therefore, this study proposes the following hypotheses H5.

**H5.**  *The extent to which an individual’s belief in their ability to perform their job adequately and deal with a wide range of tasks, known as role-breadth self-efficacy, has a negative moderating effect on the relationship between decreased self-control and employees’ positive assertive actions at work. This means that the higher an employee’s role-breadth self-efficacy, the less likely decreased self-control will have a negative effect on their performance of positive assertive actions*.

Employees’ constructive deviance is a widely acknowledged beneficial behaviour for both organisations and their members. Organisations seek employees who are capable of taking the initiative and embracing change, whilst employees value colleagues who challenge the status quo to seek additional resources and assistance for themselves or the organisation as a whole. However, the act of engaging in constructive deviance is internal and requires employees to possess additional self-control resources in order to manage “controlling self-fatigue [75]” against “assisting both the leader and the organisation [76]”. In high uncertainty job situations, where there is a lack of defined role expectations, employees can experience a lack of command over their work, leading to tension, psychological insecurity, and a resultant negative effect [77]. This experience can further deplete self-control resources. However, individuals with greater self-efficacy in their broad range of job responsibilities display enhanced psychological resilience and confidence in deviating from organisational norms and are more disposed towards constructive deviance [78]. Furthermore, employees with higher role-breadth self-efficacy may feel responsible beyond their work, which enables them to resist the negative emotions correlated with hierarchical ambivalence. Consequently, they can persist in employees’ constructive deviance. This study proposes the following hypothesis H6.

**H6.**  *The width of one’s role-breadth self-efficacy has a negative moderating effect on the indirect impact of leader–member exchange ambivalence on employees’ constructive deviance. This means that the connection between ambivalent superior–subordinate relationships and employees’ constructive transgressive behaviours through ego depletion is less intense when one’s role-breadth self-efficacy is high*.

### 2.2. Research Model

**Figure 1:**
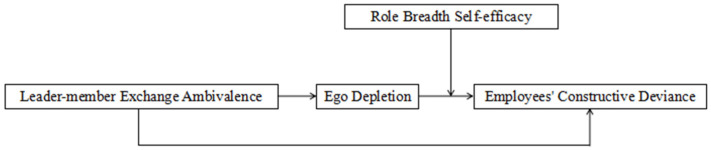
Theoretical framework.

Self-control Resource Theory posits that self-control is a finite resource, and one action’s execution depletes the resources required for another action [47]. Superior–subordinate relationships can be categorised based on varying levels of proximity and quality as a result of limited time and resources [79]. When employees perceive conflicting relationships between their superiors and subordinates, they undergo cognitive dissonance and are engulfed in negative emotions such as distrust. This fuels their perception of burnout, depletes their self-control resources, and leads to severe ego depletion [80]. Following this, employees respond by reducing the resources available to carry out other actions, such as employees’ constructive deviance. Employees with a high level of role-breadth self-efficacy are capable of replenishing the resources that are depleted due to self-control. This, in turn, lessens the inhibitory impact of ego depletion on employees’ constructive deviance exhibited by employees. Figure 1 illustrates the mechanism of influence of leader–member exchange ambivalence on employees’ constructive deviance, as deduced from the analyses presented above.

## 3. Method

The data collection method used in this study was a questionnaire. The questionnaire employed well-established scales used in authoritative journals both domestically and internationally. The questionnaire was distributed and collected online. This study randomly collects data from employees of companies in China in a variety of industries including internet, finance, IT, engineering and services. In order to reduce homophily bias, this study used two time points to collect data with a two-week interval. At time point T1, information on subjects’ personal information, leader–member exchange ambivalence, and ego depletion and control variables were collected, with 372 questionnaires distributed and 349 returned; two weeks later, a second questionnaire was distributed to subjects who had completed the questionnaire for the first time to collect information on employees’ constructive deviance, with 342 questionnaires distributed and 338 returned. In both questionnaires, subjects were required to fill in “initials + last 4 digits of mobile phone number” to ensure accuracy of questionnaire matching. After eliminating invalid questionnaires with too many repetitions and questions that did not pass the test, 332 questionnaires were validly matched, and the effective recovery rate of the questionnaires was 86.56%. In terms of gender, 44% were male and 56% female; in terms of age, 57.20% were aged 25 and under, 35.80% were aged 26–35, 3.90% were aged 36–45 and 3% were aged 45 and over. In terms of marital status, 83.40% were unmarried and 16.60% were married. In terms of education, 0.90% had completed junior high school or less, 1.50% had completed high school or junior college, 1.80% had completed junior college, 56.30% had completed a bachelor’s degree and 39.50% had completed a master’s degree or higher. In terms of time spent with unit leaders, less than one year accounted for 42.50%, 1–3 years—36.10%, 3–5 years—10.80%, 5–10 years—5.70% and more than 10 years—4.80%.

This study used mature scales from major journals at home and abroad, all of which had high reliability and validity. The English scales were translated into Chinese according to standard translation and back-translation procedures, and relevant scholars and colleagues in the field were asked to proofread the scales, and finally the measurement items for formal research were determined. All scales in this study were scored on a 5-point Likert scale ranging from 1 to 5 for “strongly disagree” to “strongly agree”. Additionally, the study utilised established scales to design the questionnaire. Upon analysis of the collected data, it was confirmed that the reliability of each scale was good, with Cronbach’s coefficients exceeding 0.8.

Leader–member exchange ambivalence was measured using a 7-item scale developed by Lee et al. [22], with representative items such as “I have ambivalent thoughts: sometimes I think I have a very good working relationship with my supervisor and sometimes I don’t”, with a Cronbach’s alpha coefficient of 0.884.

Ego depletion was measured using a 5-item scale developed by Lin and Johnson [81], with representative items such as “I feel exhausted”, with a Cronbach’s alpha coefficient of 0.845.

Role-breadth self-efficacy was measured using a 7-item scale developed by Parker et al. [82], with representative items such as “Designing new procedures for my area of work”, with a Cronbach’s alpha coefficient of 0.883.

Employee constructive deviance was measured using a 9-item scale developed by Galperin [10], with representative items such as “disobeying my supervisor’s instructions to work more efficiently”, and a Cronbach’s alpha coefficient of 0.900.

Based on existing research, gender, age, marital status, education and time spent working with the leader of the organisation were selected as control variables.

In this study, SPSS 27 and AMOS 27 were used to analyse the sample data. Firstly, AMOS 27 was used to conduct a validated factor analysis and common method bias test for the four variables of this study, namely ambivalent experience of superior–subordinate relationships, ego depletion, role-breadth self-efficacy and employees’ constructive deviance; secondly, the data were statistically analysed for descriptiveness and correlation using SPSS 27; and finally, SPSS 27 and PROCESS were used to test the mediation and moderation of the research hypotheses.

## 4. Results

In order to examine the construct differentiation of the variables, this study used AMOS 27 to perform a validation factor analysis of the models constructed for the leader–member exchange ambivalence, ego depletion, role-breadth self-efficacy and employees’ constructive deviance and to compare the fitted indicators. According to the results in Table 1, the four-factor model had the best fit (χ2/df = 1.690, CFI = 0.954, TLI = 0.950, RMSEA = 0.046, SRMR = 0.043). Accordingly, the four variables were shown to have good discriminant validity.

The data in this study were statistically analysed using SPSS 27 for descriptive and correlational purposes, and the results are presented in Table 2. Leader–member exchange ambivalence was positively correlated with ego depletion (r = 0.80, *p* < 0.001), ego depletion was negatively correlated with employees’ constructive deviance (r = −0.64, *p* < 0.001), and leader–member exchange ambivalence was negatively correlated with employees’ constructive deviance (r = −0.71, *p* < 0.001).

In this study, the research hypotheses were tested using hierarchical regression analysis using SPSS 27, and the results are presented in Table 3. Model 2 showed that leader–member exchange ambivalence had a significant negative effect on employees’ constructive deviance (b = −0.779, *p* < 0.001), and Hypothesis 1 was supported. Model 1 showed a significant positive effect of leader–member exchange ambivalence on ego depletion (b = 0.844, *p* < 0.001), and Hypothesis 2 was supported. Model 3 showed that ego depletion significantly and negatively influenced employees’ constructive deviance (b = −0.747, *p* < 0.001), and Hypothesis 3 was supported. Model 4 shows that the dependent variable, employees’ constructive deviance, regresses on both the independent variable, leader–member exchange ambivalence, and the mediating variable, ego depletion, with a significant coefficient on leader–member exchange ambivalence (b = −0. 578, *p* < 0.001) and a significant coefficient for ego depletion (b = −0.238, *p* < 0.001), and that the effect of leader–member exchange ambivalence is attenuated by the presence of ego depletion. This suggests that ego depletion partially mediates the relationship between leader–member exchange ambivalence and employees’ constructive deviance, and Hypothesis 4 is supported. Next, the study used PROCESS to test the mediating role, with a bootstrapped repeated sample size of 5000. The results showed in Table 4 that the direct effect of leader–member exchange ambivalence on employees’ constructive deviance was −0.578, with SE = 0.050 and a 95% confidence interval of CI = [−0.480,−0]. When the sense of role-breadth self-efficacy was not included in the model analysis, the indirect effect of conflicting experiences of superior–subordinate relationships on employees’ constructive deviance was −0.201, SE = 0.048, 95% confidence interval CI = [−0.304,−0.118]; and the coefficient of the influence of leader–member exchange ambivalence on employees’ constructive deviance was still significant after adding the variable of ego depletion. The results suggest that ego depletion partially mediates the relationship between leader–member exchange ambivalence and employees’ constructive deviance.

**Figure 2:**
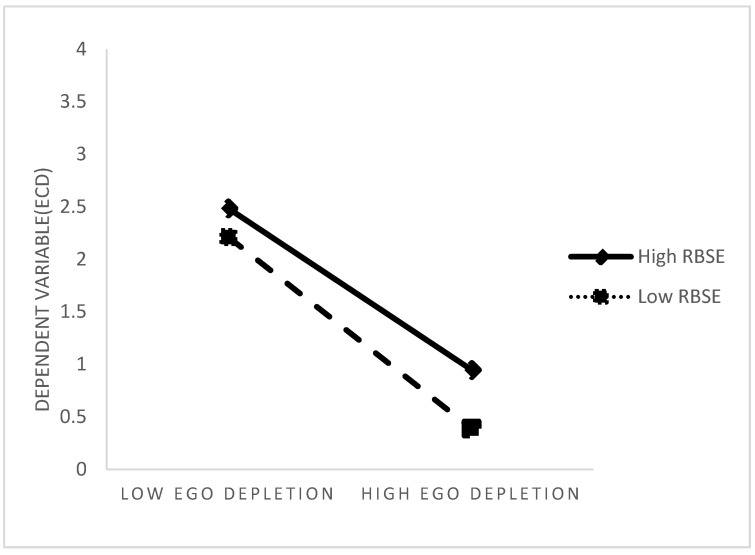
Moderating effects of role-breadth self-efficacy.

To test the moderating effect of role-breadth self-efficacy on the relationship between ego depletion and employees’ constructive deviance, the mediating and moderating variables were first centred, and the centred mediating and moderating variables were multiplied to obtain the interaction term, and then a hierarchical regression test was conducted. The results, as shown in Model 6 of Table 3, showed that after controlling for the main effects of ego depletion and role-breadth self-efficacy, the interaction of both ego depletion and role-breadth self-efficacy had a significant effect on employees’ constructive deviance (b = −0.113, *p* < 0.001, ∆R2 = 0.015), suggesting that role-breadth self-efficacy had a significant moderating effect on the relationship between ego depletion and employees’ constructive deviance, supporting Hypothesis 5. The moderating effect was significant, supporting Hypothesis 5. Further simple slope analysis was carried out to create an interaction plot with the moderating variable role-breadth self-efficacy divided into high and low levels (means plus or minus one standard deviation, respectively). As shown in Figure 2, at low levels of role-breadth self-efficacy, ego depletion was a significant negative predictor of employees’ constructive deviance (b = −0.912, *p* < 0.001); at high levels of role-breadth self-efficacy, ego depletion was a significant negative predictor of employees’ constructive deviance (b = −0.769, *p* < 0.001). The above results suggest that the negative relationship between employee ego depletion and constructive deviance is weaker at high levels of role-breadth self-efficacy.

In this study, the PROCESS method was utilised to examine the mediating effect with moderation. The findings unveiled a moderated mediation effect index of −0.049 with a standard error of 0.018 and 95% confidence interval of CI = [−0.080, −0.010] (excluding 0). The results demonstrated that the role-breadth self-efficacy served as a moderator in the association among leader–member exchange ambivalence, ego depletion, and employees’ constructive deviance. The outcomes are reinforced by the data presented in Table 5. When employees’ role-breadth self-efficacy is low, the indirect effect of ego depletion on their constructive deviance is −0.130, with a standard error of 0.041 and a 95% confidence interval of CI = [−0.219, −0.062] (not including 0). Conversely, when employees’ role-breadth self-efficacy is high, the indirect effect of ego depletion on their constructive deviance is −0.209, SE= 0.044, 95% confidence interval CI = [−0.295, 0.125] (not including 0); there is a significant difference in the mediation effect of ego depletion between high and low levels of role-breadth self-efficacy, with a difference estimate of −0.079 and SE = 0.028. The 95% confidence interval is CI = [−0.129, −0.017 (not including 0)]. The findings from the aforementioned analyses corroborate hypothesis H_6_, which posits that self-efficacy regarding one’s role breadth has a noteworthy moderating impact on how conflicting experiences within superior–subordinate relationships affect constructive deviance via ego depletion. In other words, high self-efficacy regarding one’s role breadth amplifies the indirect effect of leader–member exchange ambivalence on employees’ constructive deviance through ego depletion.

## 5. Discussion

This study examines the impact of leader–member exchange ambivalence on employees’ constructive deviance through ego depletion. It also investigates the moderating effect of role-breadth self-efficacy. The results of the research hypotheses testing will be addressed in this subsection.

The first is about the direct effect of the leader–member exchange ambivalence on employees’ constructive deviance. Based on the resource limitation principle of self-control resource theory, this study proposes negative hypotheses on the relationship between the two. The study verifies the negative correlation between leader–member exchange ambivalence and employees’ constructive deviance through hierarchical regression analysis. The statistical analysis revealed a significant negative correlation (r = −0.779, *p* < 0.001) between employees’ contradictory experience of superior–subordinate relationships and their tendency to engage in constructive transgressive behaviour. This finding supports the perspectives of self-regulation theory and resource conservation theory. Employees who experience ambivalent relationships with their superiors need to expend psychological resources to manage this ambivalence. Due to the limited nature of these resources, one behaviour may take up the resources of another. Employees may not have access to sufficient psychological resources, such as psychological ownership and psychological security, to engage in constructive transgressive behaviours. Organisations may encounter management problems due to individual obstacles, such as internal conflicts arising from superior–subordinate relationships, which can hinder effective change implementation.

The second is the mediating role of ego depletion. Ego depletion is the process by which an individual’s self-control activities result in the depletion of psychological energy, leading to a low-energy state. This study proposes a positive hypothesis on the leader–member exchange ambivalence and ego depletion, based on the finiteness principle of self-control resource theory. The empirical evidence demonstrates that ambivalent experiences of hierarchical relationships positively affect ego depletion (b = 0.844, *p* < 0.001). The positive effect is due to the experience of hierarchical ambivalence, which leads to a failure of self-regulation by frustrating individuals’ psychological resources and placing them in a state of weak self-control. After experiencing ego depletion, individuals tend to invest their remaining resources in core tasks, thereby engaging in fewer out-of-role behaviours [50].

Finally, the moderating effect of role-width self-efficacy is discussed. To examine individual differences in the leader–member exchange ambivalence, this paper introduces role-breadth self-efficacy as a moderator variable. Role-breadth self-efficacy is a unique variable that can either be a stable personal trait developed over time or temporarily induced by work situations. Individuals with high role-width self-efficacy are able to proactively engage in out-of-role behaviours that are beneficial to organisational development. Employees’ constructive deviance is a typical type of proactive and out-of-role behaviour. As supporters and implementers of organisational change, these individuals have the courage to challenge the status quo of work and adopt creative methods to deal with encountered problems. Therefore, even when experiencing the self-defeat caused by contradictory superior–subordinate relationships, individuals with high role-width self-efficacy were still able to self-regulate and persist in constructive transgressive behaviours (b = −0.769, *p* < 0.001). In contrast, individuals with low levels of role-breadth self-efficacy were less able to ameliorate ego depletion (b = −0.912, *p* < 0.001).

By exploring the negative factor of ambivalent experience of supervisor–subordinate relationships, the study provides a new theoretical perspective to the lack of research on the inhibitors of constructive transgressions by employees and deepens the closely related research on supervisor–subordinate relationships. The study identifies the insecure relationship between employees and supervisors that leads to the ambivalent experience of supervisor–subordinate relationships, and the cognitive pathway that focuses employees’ individual emotional triggers and motivations for proactive behaviour on their relationship with supervisors, helping to raise awareness of the importance of social network relationships in organisations. In addition, the study adopts the Self-Control Resource Theory to clarify the reasons for the inhibitory effect of the ambivalent experience of the superior–subordinate relationship on employees’ constructive transgressive behaviours as well as their psychological processes, thus extending the scope of the Self-Control Resource Theory. Based on social cognitive theory, the study breaks through the previous research’s interpretation of the motivational path between the ambivalent experience of superior–subordinate relationships and employees’ work outcomes and clarifies that role-breadth self-efficacy can be used as a boundary condition to alleviate the negative effects of the ambivalent experience of superior–subordinate relationships. The study explored the important factors of individual responses to the ambivalent experience of superior–subordinate relationships at the individual trait level, which responded to the research perspective of Huang et al. [83].

## 6. Conclusions

Based on self-control resource theory and social cognitive theory, this investigation explores how ambivalent experiences in superior–subordinate relationships impact employees’ constructive deviance through ego depletion. Additionally, the study evaluates how role-breadth self-efficacy moderates the relationships outlined above. The research enhances comprehension of the ambivalent experience of the superior–subordinate relationship and its impact on employees’ constructive deviance through direct and indirect mechanisms such as ego depletion and role-breadth self-efficacy. The 332 employee participants from various industries nationwide ensure the study’s generalisation and representation.

The study’s findings demonstrate that the ambivalent experience of the superior–subordinate relationship has an adverse impact on employees’ constructive deviance. As the experience decreases over time, a fluctuation trend is observed wherein the employees’ constructive deviance shows a low, medium, or high frequency. Leader–member exchange theory and the group involvement model were employed to illustrate that the correlation between leadership downward influence tactics and employees’ aiding behaviours relied more heavily on the standard of the leader–member exchange relationship. The role of leader–member exchange ambivalence influencing employees’ proactive behaviours was demonstrated. It was found that the effects of such relationships varied among employees based on their perceptions of these relationships.

In addition to the direct impact of the ambivalent experience of superior–subordinate relationships on employees’ constructive deviance, it has been noted that this ambivalent experience also indirectly affects their constructive deviance through the effective mechanism of ego depletion. Ego depletion partly mediates the correlation between the ambivalent experience of superior–subordinate relationships and the constructive deviance of staff. Inconsistency in such relationships can lead to emotional fluctuations, creating insecurity among employees, which can consequently result in frustration, anxiety, and other negative psychological outcomes. Employees who experience variability in their relationships with superiors often experience inner conflicts, which can consume significant amounts of their self-control resources. Research shows that employees who lack adequate self-control find it challenging to behave effectively and struggle to allocate adequate energy and resources towards beneficial, extra-role behaviours for their organisation and colleagues.

Incorporating role-breadth self-efficacy’s moderating effect into employees’ cognitive responses significantly increased constructive deviance among employees who experience ambivalent subordinate relationships. Role-breadth self-efficacy involves employees’ perceptions of completing extra-role tasks, while constructive deviance refers to employee-initiated behaviours with extra-role qualities. Only employees with a high level of role-breadth self-efficacy can employ adequate self-confidence to renew depleted energy and avoid self-wasting, enabling them to fully utilise psychological and material resources to engage in constructive deviance. Employees with high role-breadth self-efficacy are able to handle negative emotions arising from contradictory superior–subordinate relationships and make constructive deviance using their confidence and sense of mission. This assists in challenging the status quo and facilitating organizational reform. Similarly, the occurrence of constructive deviance is negatively affected to some extent for employees with high role-breadth self-efficacy due to leader–member exchange ambivalence and resulting ego depletion. This study confirms the adverse effect of employees’ emotional experience on their constructive deviance. Such effects are often ignored by enterprises, but this research has significant theoretical implications and provides valuable practical insights for enterprise management practices.

This study has implications for the management of organisations. The slow pace of progress in modern organisations is linked to their serious internal depletion. This depletion includes material and social factors, particularly conflicts arising from superior–subordinate relationships within the organisation’s social network. Organisational issues stem from a depletion of mental resources in individual organisations resulting from lack of understanding, cooperation, and other factors. Improved organisational effectiveness can be achieved only by reducing internal individual resource loss and transforming resources into organisational growth potential. This will truly remove obstacles to change and inject new vitality into long-term development. Due to the adverse impact of inconsistent superior–subordinate relationships on employees’ constructive deviance, it is essential that all parties in the enterprise social network emphasise the significance of such relationships for the stable development and sustainable innovation of the business. This underscores the importance of effective human resource management and the creation of a robust enterprise culture.

Enterprise level. Firstly, companies with a formal hierarchical structure should recruit employees with a strong sense of self-efficacy towards their role’s breadth. These enterprises should also enhance their corporate culture training to improve staff identification with the organization and increase employees’ sense of self-efficacy towards their role’s breadth. By doing so, staff can invest more resources into the organization’s development and construction. Additionally, businesses ought to enhance their management of effectiveness, address the prevalent internal attrition issues and work towards establishing a positive work environment. Additionally, this study investigates the inhibiting factors that impede employees’ productive nonconformist actions through the lens of self-depletion. This approach can swiftly and accurately assist companies in determining the causes of resistance to change and aid in reducing individual depletion of self-control resources caused by conflicting superior–subordinate relationships. This, in turn, can decrease the overall energy depletion within the organization. Finally, organisations should enhance staff training to comprehend ambivalence and to enhance leader consistency and efficacy in team communication. Rational utilisation of the link between leaders and employees, mitigating conflicts among subordinates and promoting the fundamental principle of “vertical cooperation”, can achieve a rational distribution of resources, maximised efficacy and sustainable development.

Leader Level. This study seeks to determine how the occurrence of leader–member exchange ambivalence influences employees’ constructive deviance in practical settings, offering leaders guidance in managing their relationships with subordinates. The research highlights that the ambiguous nature of superior–subordinate relationships hinders the emergence of beneficial boundary violations. This indicates that leaders need to ensure that their own actions and language are consistent to reduce instances of subordinates doubting the quality of such relationships, thus alleviating any ambivalence felt by them. Meanwhile, leaders ought to strive for a reasonable degree of fairness when distributing resources in order to minimise relational comparisons among workers and prevent the negative environment created by cutthroat competition. Employees must be authorised accordingly and provided with adequate motivation to boost their scope of responsibilities’ self-efficacy, allowing them to make positive modifications to the organisation with ample self-assurance. Simultaneously, leaders ought to consider the emotional management of employees and offer more humane treatment to subordinates. Furthermore, they must decrease the significance of subordinate relationships and redirect employees’ attention, prompting them to uncover meaning in their work. By assisting them in managing the conflicting state of being subordinate and attaining affirmative work results, leaders can facilitate positive outcomes.

At the employee level, objective evaluations should be prioritised. A positive mindset is essential, and it is important to acknowledge the inherent contradiction in superior–subordinate relationships. Uncertainties within these relationships should be tolerated, and ways to maintain positivity should be practised to transform this into motivation for work, even during positive and negative experiences. The study indicates that employees need to develop skills in managing upwards and communicating effectively with their leaders to address conflicts. It also highlights the importance of employees maintaining a sufficient level of self-control resources to meet the organisation’s needs at the appropriate time. Only with the support of all individuals in the organisation can employees engage in constructive deviance voluntarily and without fear of risk. This fosters synergy within the group and ultimately promotes organisational effectiveness.

Despite the significant theoretical and practical implications of this study, there are several shortcomings that require improvement in future research. Initially, while the Employee Constructive Deviance Scale employed in this study followed a rigorous back-translation method, there are still restrictions in using a scale developed in a Western context directly in a Chinese context. Further research may be necessary to refine the scale, taking into account the unique organisational culture and environment in China, to enhance its precision and validity. Additionally, respondents may display social desirability biases when evaluating themselves, potentially inflating their self-assessments to cater to societal expectations. Future studies could use a combination of self-report and evaluations from multiple sources to enhance the study’s accuracy. Additionally, situational factors, such as moderating focus, could be introduced as moderating variables. Further research could investigate the impact of such factors on the progression from conflicting experiences of superior–subordinate relationships to employees’ constructive deviance.

## Figures and Tables

**Table 1:** Results of confirmatory factor analysis (*n* = 332).

Model	χ2/df	RMSEA	SRMR	CFI	TLI
Five-factor model (LMXAS;ED;RBSE;ECD;CMV)	1.569	0.041	0.037	0.965	0.959
Four-factor model (LMXAS;ED;RBSE;ECD)	1.690	0.046	0.043	0.954	0.950
Three-factor model (LMXAS + ED;RBSE;ECD)	1.769	0.048	0.046	0.949	0.944
Two-factor model (LMXAS + ED + RBSE;ECD)	3.945	0.094	0.102	0.802	0.786
One-factor model (LMXAS + ED + RBSE + ECD)	4.071	0.096	0.104	0.793	0.776

Note. N = 332. LMXAS = leader–member exchange ambivalence; ED = ego depletion; RBSE = role-breadth self-efficacy; ECD = employees’ constructive deviance. CMV = common method variance.

**Table 2:** Standard deviation, mean and correlation coefficient of variables.

Variable	M	SD	1	2	3	4	5	6	7	8	9
1.sex	1.560	0.497	—								
2.age	1.530	0.714	−0.026	—							
3.mar	1.170	0.372	0.052	0.625 **	—						
4.edu	4.320	0.674	−0.112 *	−0.137 *	−0.115 *	—					
5.time	1.940	1.094	−0.058	0.650 **	0.558 **	−0.151 **	—				
6.LMXAS	3.086	0.806	0.113 *	−0.046	−0.002	−0.070	−0.061	(0.884)			
7.ED	2.996	0.789	0.100	−0.067	−0.008	−0.058	−0.054	0.864 **	(0.845)		
8.RBSE	3.169	0.802	−0.128 *	0.116 *	0.017	0.040	0.082	−0.362 **	−0.457 **	(0.883)	
9.ECD	2.671	0.728	−0.059	−0.002	−0.006	0.056	−0.010	−0.855 **	−0.803 **	0.412 **	(0.900)

Note. N = 332; * *p* < 0.05, ** *p* < 0.01. The value in brackets is the internal consistency coefficient of the scale.

**Table 3:** Hierarchical regression analysis results.

Variable	ED	ECD				
	Model 1	Model 2	Model 3	Model 4	Model 5	Model 6
Sex	0.004	0.048	0.027	0.049	0.034	0.030
Age	−0.055	−0.042	−0.077	−0.056	−0.085	−0.083
Mar	0.023	0.068	0.070	0.074	0.078	0.073
Edu	0.001	−0.008	0.005	−0.008	0.004	0.010
Time	0.018	−0.023	−0.002	−0.019	−0.003	0.004
LMXAS	0.844 ***	−0.779 ***		−0.578 ***		
ED			−0.747 ***	−0.238 ***	−0.720 ***	−0.707 ***
RBSE					0.061 ***	0.079 ***
ED × RBSE						−0.113 ***
R2	0.748	0.735	0.649	0.752	0.652	0.667
∆R2	0.729	0.017	0.643	0.017	0.004	0.015
F	161.030 ***	150.459 ***	100.1326 ***	140.353 ***	86.906 ***	80.904 ***
∆F	941.642 ***	895.039 ***	594.972 ***	21.837 ***	8.99 **	14.168 ***

Note. *** *p* < 0.001, ** *p* < 0.01.

**Table 4:** Analysis of mediation effect of bootstrap.

	Effect Value	Boot SE	LLCI	ULCI
Indirect effect	−0.201	0.048	−0.304	−0.118
Direct effect	−0.578	0.050	−0.641	−0.480

**Table 5:** Mediating effects with regulation.

Independent	Independent			Index of Moderated Mediation	
	Moderator	Effect	(CI)	Index	(CI)
ECD	Low RBSE (−1SD)	−0.130	[−0.219, −0.062]	−0.049	[−0.080, −0.010]
	High RBSE (+1SD)	−0.209	[−0.295, 0.125]		
	Difference (high–low)	−0.079	[−0.129, −0.017]		

## Data Availability

The data that support the findings of this study are available from the corresponding author upon reasonable request.
